# Adenosine Deaminase (ADA)-Deficient Severe Combined Immunodeficiency Unmasked by Persistent Lymphopenia and Prolonged Severe SARS-CoV-2 Infection in a Three-Week-Old Neonate

**DOI:** 10.7759/cureus.57697

**Published:** 2024-04-06

**Authors:** Zaineb Benslimane, Funda Cipe, Khurshid Khan, Maged N Nabawi, Anasalwogud Abdelmogheth

**Affiliations:** 1 Pediatrics, Al Qassimi Women's and Children's Hospital, Sharjah, ARE; 2 Pediatric Allergy/Immunology, Al Qassimi Women's and Children's Hospital, Sharjah, ARE; 3 Pediatric Intensive Care Unit, Al Qassimi Women's and Children's Hospital, Sharjah, ARE

**Keywords:** lymphopenia, pediatrics, sars-cov-2, severe combined immunodeficiency, ada deficiency, case report

## Abstract

Adenosine deaminase (ADA) deficiency, an autosomal recessive variant, is the second most common form of severe combined immunodeficiency (SCID). We report a unique case of a three-week-old neonate who presented with prolonged and severe SARS-CoV-2 infection associated with persistent lymphopenia, subsequently revealing ADA-deficient SCID. He presented with mild and insidious symptoms, and then his clinical condition rapidly deteriorated. He required ICU admission and mechanical ventilation and developed multiple co-infections including opportunistic pathogens. Flow cytometry and whole exome sequencing diagnosed ADA-deficient SCID.

This case highlights the importance of recognizing primary immunodeficiency disorders in children who consistently display lymphopenia and experience prolonged opportunistic and viral infections. Detecting lymphopenia should prompt consideration of SCID, serving as a straightforward and cost-effective screening approach, particularly in nations such as the United Arab Emirates where T-cell receptor excision circles (TRECs) are not part of newborn screening protocols.

## Introduction

Severe combined immunodeficiency (SCID) is a rare life-threatening primary immunodeficiency disorder (PID) that causes early death due to profound impairment of T and B cell functions. Adenosine deaminase (ADA) deficiency, an autosomal recessive variant, is the second most common form of SCID. Typical clinical presentation includes infants with failure to thrive and severe or opportunistic infections [1,2]. We report a unique case of a three-week-old neonate who presented with insidious initial symptoms and then developed prolonged and severe SARS-CoV-2 infection associated with persistent lymphopenia, subsequently revealing ADA-deficient SCID.

## Case presentation

Patient information

A three-week-old term male neonate from Afghanistan presented to the Emergency Department with complaints of mild dry cough and nasal congestion for two weeks, associated with decreased oral intake for one day. There was no history of sick contact, fever, dyspnea, diarrhea, or dermatitis. He was born to a mother who had six pregnancies out of which four live births (G6P4) at 37 weeks + 4 days after an uncomplicated pregnancy and a normal delivery with a birth weight of 3.16 kg. His parents were first-degree relatives. Although no immunodeficiency was documented in the family, two male siblings born at full term without complications succumbed to fatal respiratory problems at the age of three months. He was exclusively breastfed and up to date in his vaccination. He received the mandatory hepatitis B and BCG vaccines after birth as per the local immunization program in the United Arab Emirates (UAE).

Clinical findings

On admission, the baby was hemodynamically stable and afebrile. He only displayed mild tachypnea and mild subcostal retractions. He was maintaining saturation on room air and his chest auscultation revealed few conductive sounds. His CBC showed leukopenia and lymphopenia while his CRP was 3.50 mg/dL (Table 1). A SARS-CoV-2 nasopharyngeal swab for PCR was collected. 

**Table 1:** Laboratory values upon admission. CRP: C-reactive protein

	Result	Normal values
White Blood Cell Count	2250/mcL	4500-11000/mcL
Absolute Neutrophil Count	1330/mcL	1000-5000/mcL
Absolute Lymphocyte Count	250/mcL	4000-10000/mcL
CRP	3.50 mg/dL	< 3.50 mg/dL

**Figure 1:**
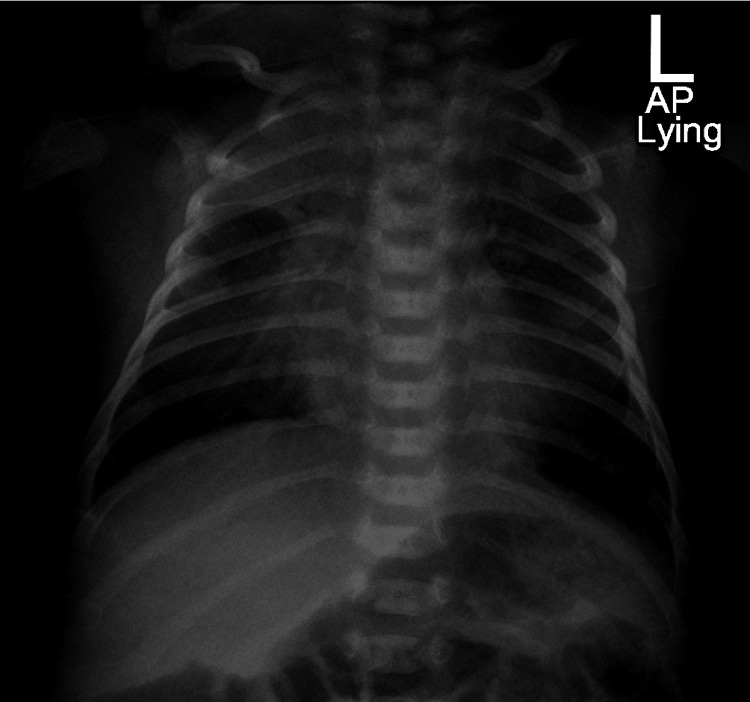
Chest X-ray (anterioposterior view) on admission showing right upper lung zone consolidation, diffuse interstitial infiltrates, and accentuated bronchovascular markings bilaterally.

A chest X-ray showed a consolidation in the right upper lung, diffuse interstitial infiltrates, and bilateral accentuated broncho-vascular markings (Figure 1). He was initially admitted to the pediatric ward as a case of pneumonia and to rule out neonatal sepsis. Cerebrospinal fluid, blood culture, and urine culture were collected and he was started on intravenous cefotaxime and penicillin. Cultures were later reported negative; however, his SARS-CoV-2 PCR came back positive and he was diagnosed with COVID pneumonia. On the second day of admission, his clinical condition started to deteriorate. He developed moderate respiratory distress and required oxygen via nasal cannula but remained afebrile. He was transferred to the Pediatric ICU where he was connected to a high-flow nasal cannula.

Timeline of the current episode

**Figure 2:**
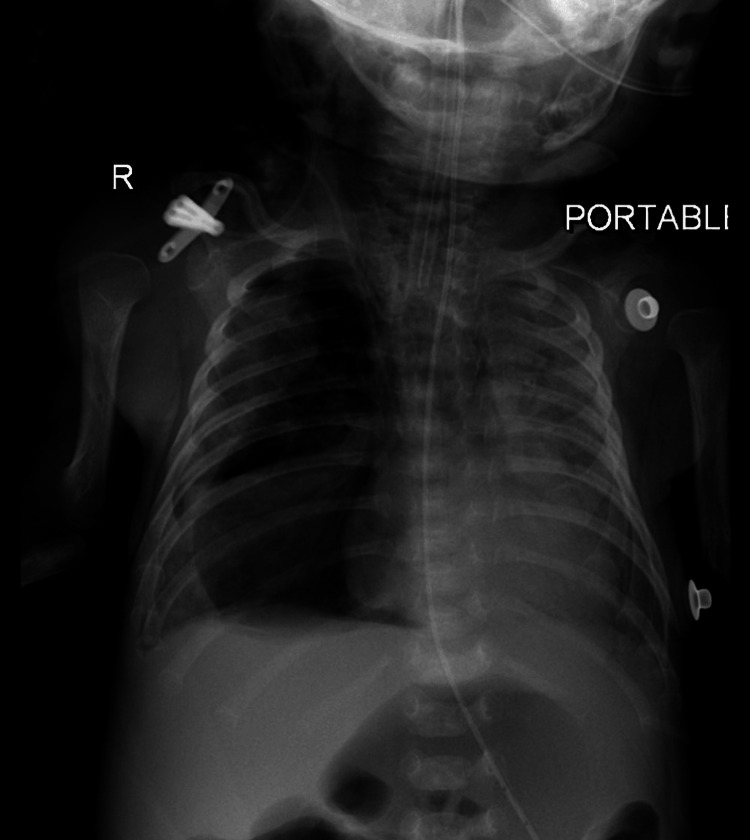
Chest X-ray (anteroposterior view) done two weeks after admission showing infiltrative lung disease and a progressive course of left lung inhomogeneous opacities.

Throughout his five weeks hospital course, his SARS-CoV-2 PCRs were persistently positive, he exhibited persistent lymphopenia with an absolute lymphocyte count reaching 250/mcL but had near-normal serum immunoglobulin levels. He suffered from progressive left lung pneumonia and infiltrative lung disease (Figure 2) and required mechanical ventilation. Microbiological investigations revealed multiple co-infections including Enterococcus feacalis pneumonia, Enterobacter cloacae urosepsis, and methicillin-resistant Staphylococcus aureus infection in the eyes and throat. There was no oral thrush, and no signs of BCGitis, but he exhibited a very poor weight gain of 240 grams over five weeks.

Diagnostic assessment

In front of this clinical picture, SCID was suspected. Flow cytometry revealed severely diminished NK-cells and T-lymphocytes while B-lymphocytes were almost not detectable (Table 2), thus diagnosing SCID T- B- NK-.

**Table 2:** Flow-cytometry results showing results in accordance with SCID T- B- NK-.

	Results	Normal values
Lymphocytes, total	0.07 tsd/uL	3.50-13.10 tsd/uL
CD3	43%	60-85%
T-lymphocytes (CD3+)	30/uL	2300-7000/uL
CD4	17%	41-68%
T-helper cells (CD3+/CD4+)	12/uL	1700-5300/uL
CD8	10%	9-23%
T-suppressor cells (CD3+/CD8+)	7/uL	400-1700/uL
CD19+	1%	4-26%
B-lymphocytes (CD19+)	1/uL	600-1900/uL
NK-cells (CD56+)	22%	3-23%
NK-cells (CD16+/CD56+)	15/uL	200-1400/uL

Therapeutic interventions

On admission, he was started on cefotaxime and penicillin in view of pneumonia and possible neonatal sepsis. Antimicrobials were then upgraded to Ertapenem as treatment and Trimethoprim/Sulfamethoxazole, Acyclovir, and Fluconazole as prophylaxis. His treatment also included regular intravenous immunoglobulin replacement, Remdesivir, and Dexamethasone for COVID-19 infection.

Follow-up and outcome interventions

**Figure 3:**
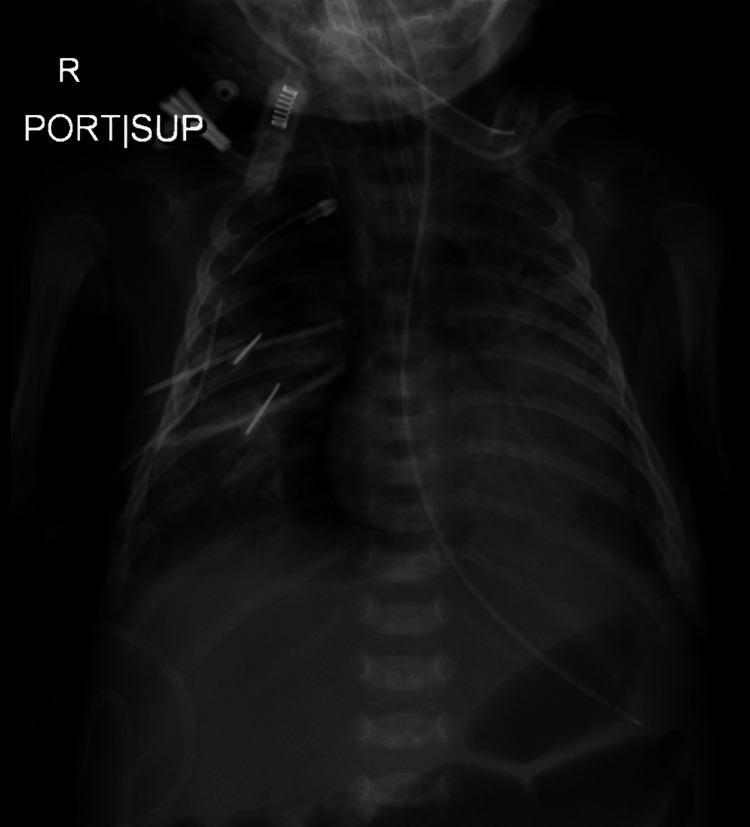
Chest X-ray (anteroposterior view) on the day of death showing pneumomediastinum and bilateral diffuse airspace shadowing.

His condition rapidly declined, leading to multi-organ dysfunction syndrome marked by acute respiratory distress syndrome (Figure 3), septic shock, and liver dysfunction. Unfortunately, he succumbed at the age of two months.

Bone marrow transplantation, the method of choice in SCID treatment [2], could not be done in view of the patient’s critical condition. Whole exome sequencing was sent and ADA-deficient SCID was genetically confirmed post-mortem. It identified a pathogenic autosomic recessive homozygous variant c.704G>Ap.(Arg235Gin) chr20/43251546 (MIM: 102700).

## Discussion

SCID is a pediatric emergency, the diagnosis starts with suspicion and the treatment should be initiated promptly, otherwise, survival beyond one-year-old is unlikely [2].

In our case, the persistent lymphopenia even with normal immunoglobulin levels, the persistent COVID-19 PCR positivity, the development of opportunistic co-infections, and the rapid clinical deterioration were all suggestive of SCID. This case shows that absolute lymphopenia can be used as a simple and inexpensive screening method for SCID in newborns and children in general [3], especially in countries where T-cell receptor excision circles (TRECs) are not included in newborn screening like the UAE. Moreover, it shows that normal immunoglobulin levels do not rule out immunodeficiency in infants less than 6 months of life as maternal immunoglobulins are still not weaned.

A systematic review of 116 articles about the severity of SARS-CoV-2 infection in children with PID involving 710 pediatric cases including 42 patients with SCID found that these children may have higher rates of prolonged infections, disease severity, ICU admission, use of mechanical ventilation, acute respiratory distress syndrome and mortality. These findings highlight the importance of preventing infections like COVID-19 in children with PID [4].

For ADA-deficient SCID, allogeneic hematopoietic stem cell transplant or autologous hematopoietic stem cell and ADA gene therapy are the only curative treatments. Enzyme replacement therapy can be used as a bridge therapy until stem cell transplantation is available [2]. Unfortunately, these therapies couldn’t be initiated for our patient as he was unstable clinically.

## Conclusions

This case highlights the importance of considering underlying PID in children exhibiting prolonged opportunistic and viral infections, such as SARS-CoV-2, particularly when accompanied by a significant family history. Absolute lymphopenia should raise the suspicion of SCID in children. This point holds great importance especially in countries where TRECs are not included in the newborn screening. Early diagnosis and treatment are crucial in improving outcomes of bone marrow transplant and saving the life of SCID patients.
